# P-273. Risk Factors for 90-day Mortality Among Patients With Stenotrophomonas maltophilia Infection: A Retrospective Multicenter study

**DOI:** 10.1093/ofid/ofae631.477

**Published:** 2025-01-29

**Authors:** Anood Al Qura'an, Wilmer Salazar, Zaid Al khouri, Ruhul Munshi, Patricia Pichilingue Reto, Paulette Pinargote

**Affiliations:** Louisiana State University Health Sciences Center Shreveport, Shreveport, Louisiana; Universidad Catolica Santiago de Guayaquil, Shreveport, Louisiana; Louisiana State University Health Sciences Center Shreveport, Shreveport, Louisiana; LSU Health Shreveport, Shreveport, Louisiana; Louisiana State University Health Sciences Center Shreveport, Shreveport, Louisiana; Louisiana State University Health Services Center Shreveport, shreveport, Louisiana

## Abstract

**Background:**

Stenotrophomonas maltophilia is a leading cause of mortality in critically ill and immunocompromised patients. This study aimed to characterize risk factors for mortality and evaluate if combination therapy or resistance patterns affect mortality in patients with S. maltophilia infections.

**Methods:**

We conducted a retrospective, observational study at two academic medical centers in the United States including patients aged ≥ 18 years with positive S. maltophilia cultures between January 2019 and March 2024. Logistic regression analysis was performed to identify independent risk factors for 90-day mortality. A sub analysis for patients with pneumonia was performed. Standard of care was defined as monotherapy with trimethoprim-sulfamethoxazole (SXT) or levofloxacin.

**Results:**

A total of 128 patients were included, median age was 66 years; 60.9% were male. 30- and 90-day mortality rates were 17.2% and 25%, respectively. The most common source of infection was pneumonia (52, 40.6%), which also had the highest mortality rates (32.7%, 46.2%) when compared to other sources of infection. Although non-survivors had higher rates of COPD (p=0.04), prior organ transplants (p=0.002), prior steroid use (p=0.03), and higher ICU admission rates (p< 0.001), they were not found to be independent risk factors for mortality.

Risk factors for 90-day mortality include neutropenia (OR 12.237, p=0.047), polymicrobial infection (OR 7.93, p=0.009) and previous carbapenem use (OR 7.34, p=0.015). Enterobacterales and fungal infections were the most common co-infections.

Monotherapy (117, 91%), and resistance to STX or levofloxacin (4.69% and 10.9% respectively) were not risk factors for mortality (OR 0.9, p=0.9; OR 3.1, p=0.58). Interestingly, osteomyelitis was associated with reduced odds of 90-day mortality (OR 0.008, p=0.015). Independent risk factors for mortality in patients with pneumonia include prior carbapenem use (OR 6.54, p=0.029) and polymicrobial infection (OR 10.95, p=0.08).

**Conclusion:**

S. maltophilia infection is associated with high mortality rates, particularly in patients with neutropenia, polymicrobial infection and prior carbapenem use. Although combination therapy or resistance to standard of care was not found to impact mortality, larger-scale studies are needed.

**Disclosures:**

**All Authors**: No reported disclosures

